# ProNGF and Neurodegeneration in Alzheimer’s Disease

**DOI:** 10.3389/fnins.2019.00129

**Published:** 2019-02-22

**Authors:** Margaret Fahnestock, Arman Shekari

**Affiliations:** ^1^Department of Psychiatry and Behavioural Neurosciences, McMaster University, Hamilton, ON, Canada; ^2^Neuroscience Graduate Program, McMaster University, Hamilton, ON, Canada

**Keywords:** nerve growth factor, TrkA, p75NTR, neurotrophins, basal forebrain cholinergic neurons, survival, apoptosis, retrograde transport

## Abstract

Profound and early basal forebrain cholinergic neuron (BFCN) degeneration is a hallmark of Alzheimer’s disease (AD). Loss of synapses between basal forebrain and hippocampal and cortical target tissue correlates highly with the degree of dementia and is thought to be a major contributor to memory loss. BFCNs depend for their survival, connectivity and function on the neurotrophin nerve growth factor (NGF) which is retrogradely transported from its sites of synthesis in the cortex and hippocampus. The form of NGF found in human brain is proNGF. ProNGF binds to the NGF receptors TrkA and p75^NTR^, but it binds more strongly to p75^NTR^ and more weakly to TrkA than does mature NGF. This renders proNGF more sensitive to receptor balance than mature NGF. In the healthy brain, where BFCNs express both TrkA and p75^NTR^, proNGF is neurotrophic, activating TrkA-dependent signaling pathways such as MAPK and Akt-mTOR and eliciting cell survival and neurite outgrowth. However, if TrkA is lost or if p75^NTR^ is increased, proNGF activates p75^NTR^-dependent apoptotic pathways such as JNK. This receptor sensitivity serves as a neurotrophic/apoptotic switch that eliminates BFCNs that cannot maintain TrkA/p75^NTR^ balance and therefore synaptic connections with their targets. TrkA is increasingly lost in mild cognitive impairment (MCI) and AD. In addition, proNGF accumulates at BFCN terminals in cortex and hippocampus, reducing the amount of trophic factor that reaches BFCN cell bodies. The loss of TrkA and accumulation of proNGF occur early in MCI and correlate with cognitive impairment. Increased levels of proNGF and reduced levels of TrkA lead to BFCN neurodegeneration and eventual p75NTR-dependent apoptosis. In addition, in AD BFCNs suffer from reduced TrkA-dependent retrograde transport which reduces neurotrophic support. Thus, BFCNs are particularly vulnerable to AD due to their dependence upon retrograde trophic support from proNGF signaling and transport.

## Basal Forebrain Cholinergic Neurons in Alzheimer’s Disease

The Alzheimer’s disease (AD) brain undergoes progressive dysfunction, degeneration and loss of neurons and synapses in selective areas of the brain (Coleman and Flood, 1987; Scheff et al., 1990). Amyloid-β, a component of plaques, has long been targeted as a precipitating insult in AD, triggering a cascade that leads to tau aggregation, impaired synaptic communication and neuronal death (Hardy and Selkoe, 2002). Tau, the primary component of neurofibrillary tangles, promotes the assembly and stabilization of microtubules in the cytoskeleton. When tau is abnormally phosphorylated, it detaches from microtubules and polymerizes into soluble oligomers, straight and paired helical filaments that assemble into neurofibrillary tangles. This tau disrupts axonal transport and is thought to lead to neuronal atrophy by loss of normal tau function and/or by gain of pathological function through toxicity of phosphorylated, truncated or aggregated forms of tau (Iqbal and Grundke-Iqbal, 2008; Ittner and Götz, 2011). As the disease progresses, both tau and amyloid pathology impact significant areas of the cortex and hippocampus. Efforts aimed at determining where the pathology begins have implicated TrkA-expressing cholinergic neurons in the basal forebrain in the very early stages of the disorder (Schmitz and Nathan Spreng, 2016).

Basal forebrain cholinergic neurons (BFCNs) are extremely important for learning, memory, and attention (Baxter and Chiba, 1999). They are among the first to degenerate and the most severely affected in aging and AD (Coyle et al., 1983; Hyman et al., 1984; Whitehouse, 1998; Schmitz and Nathan Spreng, 2016), but the reasons for the selective vulnerability of these neurons is unknown. BFCN degeneration correlates strongly with the degree of dementia and with AD pathology (Wilcock et al., 1982; Coyle et al., 1983; Bierer et al., 1995; Whitehouse, 1998). The vulnerability of BFCNs in AD may be related to disrupted communication with target neurons in the hippocampus and cortex, regions that also display pathology and neurodegeneration in AD.

Basal forebrain cholinergic neurons are dependent upon the neurotrophin nerve growth factor (NGF) for survival and proper function. They do not make NGF themselves and must obtain it through retrograde transport from their targets. Older literature focused on the mature form of NGF, demonstrating that it is highly expressed in developing and adult hippocampus and cerebral cortex and is retrogradely transported from these target areas by BFCNs (Seiler and Schwab, 1984; DiStefano et al., 1992; Lapchak et al., 1993). NGF supports differentiation and survival of BFCNs *in vitro* (Hartikka and Hefti, 1988; Hatanaka et al., 1988; Friedman et al., 1993) and *in vivo* (Hefti, 1986; Williams et al., 1986; Lapchak and Hefti, 1991; Koliatsos et al., 1994). NGF increases acetylcholine (Ach) synthesis and release (Hatanaka et al., 1988; Takei et al., 1989; Lapchak and Hefti, 1991; Rylett et al., 1993; Pongrac and Rylett, 1996; Oosawa et al., 1999; Auld et al., 2001a,b) as well as activity and expression of cholinergic markers including choline acetyltransferase (ChAT; Williams and Rylett, 1990; Lorenzi et al., 1992; Koliatsos et al., 1994; Pongrac and Rylett, 1996) and vesicular Ach transporter (VAChT; Takei et al., 1997; Oosawa et al., 1999), which are decreased in AD (Bartus et al., 1982). NGF also increases expression of its own receptor, TrkA, in BFCN (Holtzman et al., 1992; Kojima et al., 1994, 1995; Li et al., 1995). Because BFCN rely on neurotrophins for their survival and function, it has been proposed that BFCN loss in aging and AD arises from lack of neurotrophic support (Appel, 1981; Hefti and Weiner, 1986; Price, 1986; Hefti et al., 1989). In fact, significant literature supports deficits in BDNF expression in AD (Fahnestock et al., 2002; Peng et al., 2005; Fahnestock, 2011) and also disruptions of NGF and its receptor, TrkA, with concomitant effects on attention, learning, and memory (Mufson et al., 1996, 2005, 2007; Counts et al., 2004; Peng et al., 2004; Perez et al., 2011; Parikh et al., 2013). However, contrary to initial hypotheses (Appel, 1981), loss of NGF expression does not occur in AD (Jetté et al., 1994; Fahnestock et al., 1996, 2001; Peng et al., 2004). We and others showed some years ago that despite normal levels of NGF mRNA expression in human brain tissue from AD subjects (Jetté et al., 1994), NGF-immunoreactive protein detected by ELISA or bioassay is increased in cortex and hippocampus and decreased in basal forebrain, suggesting that NGF-immunoreactive material accumulates in AD due to failed BFCN retrograde transport (Crutcher et al., 1993; Scott et al., 1995; Fahnestock et al., 1996; Narisawa-Saito et al., 1996). This immunoreactive material is entirely present as proNGF (Fahnestock et al., 2001). ProNGF protein is increased in BFCN target tissues both in AD (Fahnestock et al., 2001; Peng et al., 2004) and in the human tauopathy, Pick’s disease (Belrose et al., 2014). In AD, the accumulation of proNGF in cortex and hippocampus and its reduction in basal forebrain suggest a deficit in retrograde transport of proNGF leading to a lack of survival signaling and eventual neurodegeneration.

**FIGURE 1:**
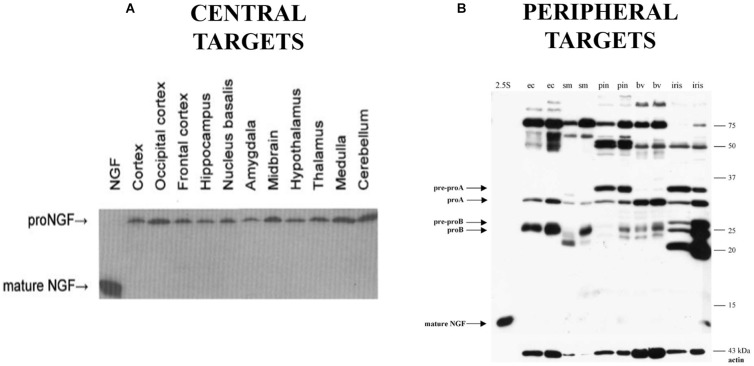
Western blot probing for NGF immunoreactivity in human brain tissue **(A)** and peripheral targets **(B)** demonstrating the presence of proNGF and the absence of mature NGF in the indicated areas (ec, external carotid; sm, submandibular gland; pin, pineal gland; bv, extracerebral blood vessels). **(A)** Reprinted from Fahnestock et al. (2001), with permission from Elsevier. **(B)** Reprinted from Bierl et al. (2005), with permission from Elsevier.

Animal models of AD further support the role of dysfunctional proNGF trafficking in AD, as the Ts65Dn mouse exhibits cholinergic degeneration and deficits in retrograde transport of proNGF (Salehi et al., 2006). However, this mouse also exhibits deficits in the NGF metabolic pathway responsible for processing proNGF to mature NGF (Iulita et al., 2014). This is consistent with an alternative hypothesis of proNGF accumulation in AD that suggests the accumulation of proNGF in AD is due to defective processing of proNGF into its mature form (Bruno and Cuello, 2006; Cuello and Bruno, 2007). This hypothesis is supported by decreases in tissue plasminogen activator and plasmin, which can process proNGF, and increases in the NGF degradative enzyme MMP-9 in MCI and in Down’s syndrome (Bruno et al., 2009a,b; Iulita et al., 2014). Both transport and processing mechanisms may be at work. However, if proNGF were processed to NGF in the normal brain, mature NGF should be detectable in normal human brain tissue, but it is not (Figure 1A; Fahnestock et al., 2001). Further, it is not clear that plasmin and MMP-9 are the endogenous proNGF/NGF processing enzymes in brain. Thus, although proNGF is acknowledged to be the form of NGF in the brain, the mechanism behind its accumulation in AD remains controversial.

## Biological Relevance of Neurotrophins

All neuronal cells require neurotrophic factors for their proper function and survival. NGF belongs to the neurotrophin family of neurotrophic factors, secreted proteins that are required for maintenance of connectivity, synaptic plasticity and survival (Maisonpierre et al., 1990; Fahnestock, 1991, 2011; Ip et al., 1992). It has been amply demonstrated both *in vitro* and *in vivo* that a deficit of neurotrophins can lead to cell dysfunction and death, and that neurotrophins can be used to rescue compromised neuronal populations from atrophy or death (Koliatsos et al., 1991; Mount et al., 1994; Rice et al., 1998; Schober et al., 1998; Klein et al., 2000). The overlapping but distinct specificities of the neurotrophins for various subsets of neurons has been proposed to explain selective neuronal impairments in particular diseases, raising the possibility that individual neurotrophins may be used therapeutically to target specific types of neurons (Hefti, 1994; Lindsay et al., 1994; Apfel, 2002; Lambiase et al., 2003; Duman and Monteggia, 2006). However, neurotrophins are not simple survival factors and can trigger cellular dysfunction and cell death under certain conditions related to their processing and receptor availability. This duality in neurotrophin function makes them critical for maintaining the balance of survival and death in the nervous system, a balance that is disrupted in AD.

## Post-Translational Processing of Neurotrophin Precursors to Produce Mature Neurotrophins

The first member of the neurotrophin family to be discovered was NGF (Levi-Montalcini and Angeletti, 1968; Levi-Montalcini, 1987). NGF supports the development, survival and function of PNS (sympathetic and sensory) and CNS (cholinergic) neurons (Levi-Montalcini and Angeletti, 1968; Thoenen and Barde, 1980; Ruit et al., 1992). Murine NGF is translated from two major alternatively spliced transcripts to produce 34 and 27 kDa prepro species (Selby et al., 1987; Fahnestock, 1991). Removal of the signal sequence reduces these translation products to proNGF species of 32 and 25 kDa (Edwards et al., 1986, 1988b; Selby et al., 1987), which occur widely (Berger and Shooter, 1977; Edwards et al., 1988b; Dicou, 1992; Seidah et al., 1996b; Chen et al., 1997; Reinshagen et al., 2000; Fahnestock et al., 2001; Bierl et al., 2005) and were initially thought to be biologically inactive (Edwards et al., 1988a; Suter et al., 1991). ProNGF can undergo further post-translational processing at both amino- and carboxyl-terminal ends to generate a mature, biologically active product of 13.2 kDa (Fahnestock, 1991). Processing of proneurotrophins may occur either intra- or extracellularly. The kallikrein γNGF, which in mouse submandibular gland is found intracellularly in a complex with NGF, processes proNGF to produce intermediate and mature forms of NGF (Greene et al., 1968; Edwards et al., 1988a; Jongstra-Bilen et al., 1989). Furin and other prohormone convertases are able to process proNGF, proBDNF, and proNT-3 to their mature forms (Bresnahan et al., 1990; Seidah et al., 1996a,b). ProNGF can also be processed extracellularly by plasmin and by matrix metalloproteases (Lee et al., 2001; Bruno and Cuello, 2006). In the CNS, proNGF is largely unprocessed (Fahnestock et al., 2001).

We now know that proNGF possesses biological activity independent from its mature isoform (Lee et al., 2001; Fahnestock et al., 2004a,b; Masoudi et al., 2009). Based on the mouse submandibular gland as a model, it was previously thought that proNGF is mainly processed into the mature form which accounts for the biological activity in most tissues (Edwards et al., 1988a; Suter et al., 1991). ELISA assays and immunohistochemistry for NGF are widely used and were thought to measure the mature 13.2 kDa NGF protein as the major form of NGF (Randolph et al., 2007). Little had been done to study NGF biosynthesis in other species or tissues, particularly CNS tissue. In 2001, we were the first to demonstrate (Fahnestock et al., 2001) that in rodent and human brain, NGF exists as 32 kDa proNGF (Figure 1A). In rodent brain there is some but very little mature NGF, while mature NGF is completely absent from human brain. The proNGF found in these tissues is not simply an intracellular precursor; it has since been accepted that secretion of unprocessed proNGF occurs from many cells and tissues, including neurons and astrocytes (Chen et al., 1997; Delsite and Djakiew, 1999; Mowla et al., 1999; Yardley et al., 2000; Lee et al., 2001; Fahnestock et al., 2004a; Bruno and Cuello, 2006), and that proNGF is the major species in both PNS (Figure 1B) and CNS tissues (Figure 1A; Fahnestock et al., 2001; Bierl et al., 2005). This distinction between the pro and mature isoforms of NGF is extremely important, as they have the potential to activate different signaling pathways, a behavior completely contingent on the receptors they bind to.

## TrkA and p75^NTR^ Receptors and Their Signaling Pathways

**FIGURE 2:**
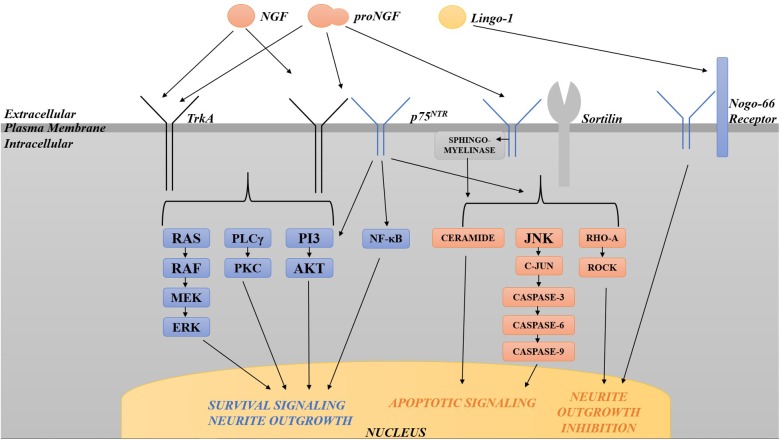
Diagram of the various pathways activated by TrkA and p75^NTR^. TrkA in isolation activates pro-survival pathways including the Ras-MAP kinase, PLCγ, and PI3 kinase/Akt pathways. p75^NTR^, when complexed with TrkA, increases pro-survival signaling through TrkA. p75^NTR^ in isolation can also signal survival via the NF-κB and PI3/Akt pathways. p75^NTR^ in isolation activates apoptosis via sphingomyelinase/ceramide, JNK, c-Jun phosphorylation and caspases 3, 6, and 9. When complexed with sortilin, p75^NTR^ binds proNGF with a higher affinity than NGF, signaling cell death through the same pathways. p75^NTR^ can also cause growth cone retraction/neurite outgrowth inhibition when complexed with the Nogo-66 receptor and its ligand Lingo-1. The balance of Trks and p75^NTR^ is crucial to the functional outcome of neurotrophin binding.

Neurotrophins are structurally related and bind similarly to two types of receptors (Figure 2). All neurotrophins bind with the same low affinity to the common neurotrophin receptor, p75^NTR^, and with high affinity to a family of type I transmembrane receptor tyrosine kinases known as Trks (tropomyosin-related kinases; Barker and Murphy, 1992; Barbacid, 1995). TrkA is the primary receptor for NGF, although there is overlap in binding specificities (Segal, 2003). Trks are generally responsible for signaling survival, differentiation, synapse strengthening, and neurite outgrowth in response to ligand (Kaplan and Miller, 2000; Miller and Kaplan, 2001; Huang and Reichardt, 2003), whereas p75^NTR^ mediates apoptosis as well as survival, synapse weakening, and inhibition/retraction of neurite outgrowth depending on its binding partner (Roux and Barker, 2002; Barker, 2004). The balance of Trks and p75^NTR^ is crucial to the functional outcome of neurotrophin binding; sufficient amounts of activated Trks, for example, can suppress apoptotic pathways activated by p75^NTR^ (Yoon et al., 1998; Segal, 2003), and p75^NTR^, when complexed with TrkA, increases NGF signaling through TrkA to enhance survival and neurite outgrowth (Epa et al., 2004). In many cell culture systems including rat oligodendrocytes, Schwann cells, embryonic BFCN, and embryonic retinal ganglion cells which express little or no TrkA, p75^NTR^ mediates apoptosis (Roux and Barker, 2002; Volosin et al., 2006). When complexed with sortilin, p75^NTR^ binds proNGF with a higher affinity than NGF, signaling cell death (Nykjaer et al., 2004). p75^NTR^ can also cause growth cone retraction/neurite outgrowth inhibition when complexed with the Nogo-66 receptor (NgR) and its ligand Lingo-1 (Bandtlow and Dechant, 2004; Barker, 2004), Whether p75^NTR^ signals cell death or cell survival depends both on the ligand and on the presence or absence of TrkA and sortilin (Barker, 2004; Ioannou and Fahnestock, 2017). As a result, maintaining the proper balance between TrkA, p75^NTR^ and sortilin is critical for neuronal function and survival.

Several intracellular signaling pathways are activated by activated Trk receptors and by p75^NTR^, including the Ras-MAP kinase, PLCγ, and PI3 kinase/Akt pathways for Trks, and the NF-κB and PI3 kinase/Akt, and the sphingomyelinase/ceramide and c-Jun N-terminal kinase (JNK) pathways for p75^NTR^ (Dobrowsky et al., 1994; Kaplan and Miller, 2000; Miller and Kaplan, 2001; Dechant and Barde, 2002; Roux and Barker, 2002; Huang and Reichardt, 2003; Segal, 2003). p75^NTR^ signaling in isolation activates apoptosis via JNK, c-Jun phosphorylation and caspases 3, 6, and 9 (Troy et al., 2002; Bhakar et al., 2003). p75^NTR^ can signal survival through activation of NF-κB (Ladiwala et al., 1998; Foehr et al., 2000; Hughes et al., 2001) or the PI3K/Akt pathway (Roux et al., 2001). Lastly, p75^NTR^ also limits neurite outgrowth via RhoA activation (Roux and Barker, 2002): p75^NTR^ null mice exhibit enhanced sympathetic and sensory sprouting (Kohn et al., 1999; Petrie et al., 2013) and hyper-innervation of the hippocampus by cholinergic neurons (Yeo et al., 1997). These signal transduction pathways downstream of both receptors have been intensely investigated in the case of NGF due to the initial assumption of the biological inactivity of proNGF. Once the biological significance of proNGF was made clear, it was found that many of the same Trk- and p75^NTR^-induced pathways are activated by proNGF.

## Biological Activity of proNGF

We and others have shown that both native and recombinant proNGF, with or without amino acid substitutions or tags, has neurite outgrowth and neuronal survival activity (Saboori and Young, 1986; Chen et al., 1997; Rattenholl et al., 2001; Fahnestock et al., 2004b; Clewes et al., 2008; Masoudi et al., 2009; Ioannou and Fahnestock, 2017). Many investigators (Lee et al., 2001; Fahnestock et al., 2004b) use mutated proNGFs that are resistant to processing into mature NGF to rule out any contribution of mature NGF. This cleavage-resistant proNGF binds to and activates TrkA and its downstream signal transducers, Erk1/2 (Fahnestock et al., 2004b; Masoudi et al., 2009) and Akt (Ioannou and Fahnestock, 2017), but with less affinity than mature NGF. Although proNGF has a lower affinity for TrkA than does NGF (Clewes et al., 2008), proNGF produces the same maximal activity as NGF (Fahnestock et al., 2004b); the low affinity is outweighed by the relative abundance of proNGF in the adult brain. In addition, endogenously expressed proNGF is similarly neurotrophic *in vivo*: transgenic mice over-expressing wild-type proNGF exhibit robust sympathetic and sensory axonal sprouting into brain parenchyma with no cell loss in peripheral ganglia (Buttigieg et al., 2007).

In contrast to these data, Lee et al. (2001) reported that proNGF exhibits apoptotic activity. Neither the many structural differences between the different cleavage-resistant proNGFs nor different expression systems or purification methods accounted for their differences in biological activity (Masoudi et al., 2009). However, cell culture conditions, which influence receptor complement, explain why some labs report apoptotic activity of proNGF whereas others report neurotrophic activity. ProNGF exhibits neurite outgrowth and survival activity on newborn rodent superior cervical ganglion neurons, which maintain relatively high TrkA levels compared to little or no p75^NTR^ (Fahnestock et al., 2004b; Masoudi et al., 2009). ProNGF is also neurotrophic for rat pheochromocytoma (PC12) cells differentiated (primed) by growth in NGF, which express high levels of TrkA vs. p75^NTR^, whereas unprimed PC12 cells (grown in serum), which express high levels of p75^NTR^ compared to TrkA, respond to proNGF with increased apoptosis (Fahnestock et al., 2004b; Masoudi et al., 2009; Ioannou and Fahnestock, 2017). Importantly, this contrasts with NGF, which is neurotrophic under both growth conditions (Ioannou and Fahnestock, 2017). Supporting the importance of relative TrkA vs. p75^NTR^ levels, apoptotic activity of proNGF *in vivo* has been shown only in situations where TrkA is missing or down-regulated and p75^NTR^ is up-regulated (Beattie et al., 2002; Harrington et al., 2004; Volosin et al., 2006; Provenzano et al., 2008). Furthermore, knocking down TrkA in PC12 cells changes proNGF’s neurotrophic activity to apoptotic activity (Ioannou and Fahnestock, 2017). Lastly, proNGF is apoptotic for TrkA-deficient PC12^nnr5^ cells but is neurotrophic for TrkA-over-expressing PC12^nnr5^-B5 cells (Ioannou and Fahnestock, 2017).

It was reported that proNGF requires intracellular proteolysis to activate TrkA (Boutilier et al., 2008), a finding that contrasts with the fact that little to no mature NGF is present in both human and rodent brains. However, the MAPK and Akt assays that were carried out to establish this occurred for 1 h, a longer time period than usual for these assays and sufficient to allow proNGF endocytosis and cleavage. In contrast, we demonstrated that cleavage-resistant proNGF robustly activates MAPK after 5 min. Furthermore, proNGF activates TrkA even when incubated for 1 h in the presence of a furin inhibitor which allows uptake but not cleavage (Masoudi et al., 2009). These results demonstrated that cleavage to mature NGF is not required for proNGF signaling and implicate proNGF as the form of NGF that normally signals and is endocytosed. Thus, proNGF binds to TrkA and is internalized without cleavage, and endocytosed proNGF activates MAPK. Furthermore, this internalized proNGF associates with signaling endosomes in PC12 cells (Di Matteo et al., 2017) and is retrogradely transported in dorsal root ganglion neurons (De Nadai et al., 2016).

In sum, proNGF can signal without cleavage, and the balance between TrkA and p75^NTR^ determines whether proNGF is neurotrophic or apoptotic. This is of critical importance because TrkA is lost in AD (Mufson et al., 1996; Counts et al., 2004), as discussed below.

## Retrograde Signaling and Transport

Axonal transport defects are common characteristics of many neurodegenerative diseases, and mutations in components of the axonal transport machinery have demonstrated that impaired axonal transport can cause neurodegeneration (Perlson et al., 2010). Lack of retrograde neurotrophic support has been postulated to cause neurodegeneration and death of BFCNs in AD (Appel, 1981). BFCN cell bodies may receive a neurotrophic signal from their distal axon terminals in the cortex and hippocampus by binding of ligand to receptors at axon terminals to produce local signals within axon terminals and retrograde signals to the cell body (Zhang et al., 2000; MacInnis and Campenot, 2002; MacInnis et al., 2003), internalization of the receptor-ligand complex and retrograde transport to cell bodies via signaling endosomes containing activated TrkA complexed with ligand (Grimes et al., 1996; Watson et al., 1999; Delcroix et al., 2003; Ye et al., 2003), or both (Campenot and MacInnis, 2003; Ascano et al., 2012). Both mechanisms of neurotrophic signaling may be compromised in AD.

Reduced proNGF signaling and transport may be due to disrupted transport machinery or to reduced TrkA, or both. In animal models, unilateral fimbrial transection, which severs cholinergic projections from the medial septum to the hippocampus, and colchicine, which disrupts axonal transport, increase hippocampal NGF-immunoreactivity but not NGF mRNA (Korsching et al., 1986; Ginn and Peterson, 1992), consistent with interrupted retrograde transport and with our findings in human brain. Reduced retrograde transport of NGF has been demonstrated in animal models of aging and disease (Cooper et al., 1994; Salehi et al., 2006), and abnormal tau phosphorylation and aggregation were shown to inhibit both anterograde and retrograde transport in the squid giant axon (Tiernan et al., 2016).

TrkA mRNA is lost in the basal forebrain of persons with AD (Mufson et al., 1996, 1997; Counts et al., 2004; Ginsberg et al., 2006). This decreases the amount of TrkA protein destined for anterograde transport to BFCN distal axon terminals (Scott-Solomon and Kuruvilla, 2018). TrkA protein levels are reduced in the cortex (BFCN terminal axons) of AD patients (Mufson et al., 1997; Counts et al., 2004; Ginsberg et al., 2006), while many studies, but not all, report no change in p75^NTR^ levels (Counts et al., 2004; Mufson et al., 2005, 2007; Ginsberg et al., 2006). Internalization and retrograde transport of mature NGF requires TrkA (Eveleth and Bradshaw, 1992; Loeb and Greene, 1993), but whether this is true of proNGF is unknown. p75^NTR^ is not required for NGF transport (Curtis et al., 1995) but may be capable of retrograde transport of NGF in the absence of TrkA (von Bartheld et al., 1996). Interestingly, the PI3K pathway may be required for TrkA-mediated retrograde transport, since inhibition of TrkA-mediated PI3K activity blocks retrograde transport of NGF in sympathetic neurons (Bartlett et al., 1998; Reynolds et al., 1998).

Recently, TrkA has been shown to bind APP, the precursor to amyloid-β, resulting in its non-pathological processing (Matrone et al., 2008a,b; Calissano et al., 2010). Thus, loss of TrkA may be an early event in AD underlying toxic amyloid buildup, consistent with the idea of basal forebrain degeneration being a possible initiating occurrence in AD. In addition, both aggregated Aβ and tau impede the bidirectional transport of TrkA in hippocampal neurons (Vossel et al., 2010). Tau reduction prevents Aβ-induced impairment of transport of TrkA but not of BDNF (Ramser et al., 2013). Thus, both Aβ and tau may regulate neurotrophin and neurotrophin receptor axonal transport.

Reduced retrograde transport of NGF has been demonstrated in animal models of aging and Down’s syndrome (Cooper et al., 1994; Salehi et al., 2006). Animal models of AD exhibit reduced TrkA as well as defective NGF retrograde transport (Cooper et al., 2001; Salehi et al., 2006). Reduced TrkA and/or retrograde transport may lead to neurodegeneration and cell death in the presence of increased proNGF (Masoudi et al., 2009; Ioannou and Fahnestock, 2017; Allard et al., 2018). Thus, multiple mechanisms may be at work to reduce the trophic support of BFCNs in AD.

## Summary

ProNGF is abundant in the CNS and can have both neurotrophic and apoptotic activities, depending on the receptor complement. ProNGF binds strongly to p75^NTR^, but it also binds to TrkA and elicits survival signaling through the MAPK and Akt pathways. ProNGF controls survival and death of NGF-dependent neurons in the basal forebrain, depending on the balance of TrkA and p75^NTR^. This is particularly important because TrkA receptors are lost in AD.

Axonal transport defects are common characteristics of many neurodegenerative diseases, and mutations in components of the axonal transport machinery have demonstrated that impaired axonal transport can cause neurodegeneration. BFCNs are uniquely dependent upon a constant supply of target-derived proNGF for their survival and function, and therefore reduced retrograde axonal transport of proNGF may cause degeneration and death of BFCNs. These neurons are crucial for learning, memory and attention, they are among the most vulnerable neurons in aging and AD, and they depend upon target-derived proNGF for their survival and function.

Multiple mechanisms may limit survival of BFCNs in AD. ProNGF accumulates in cortex and hippocampus in AD but is reduced in basal forebrain, suggesting a retrograde transport defect. The transport deficit may be the result of AD pathology or loss of TrkA, or both. The accumulation of proNGF, along with loss of TrkA, reduces retrograde survival signals and switches proNGF signaling to p75^NTR^ apoptotic pathways, thus initiating a neurodegenerative cascade. Rescue of axonal transport of signaling endosomes carrying cargoes such as BDNF, which supports motor neurons, ameliorates disease progression in a mouse model of ALS (Kieran et al., 2005). Thus, restoring retrograde transport of proNGF, for example by increasing TrkA levels, may inhibit disease progression in AD. Understanding proNGF biological activity and metabolism will aid in designing treatments to prevent degeneration of BFCNs.

## Author Contributions

All authors listed have made a substantial, direct and intellectual contribution to the work, and approved it for publication.

## Conflict of Interest Statement

The authors declare that the research was conducted in the absence of any commercial or financial relationships that could be construed as a potential conflict of interest.
